# Event DAS-444Ø6-6 soybean grown in Brazil is compositionally equivalent to non-transgenic soybean

**DOI:** 10.1080/21645698.2016.1184815

**Published:** 2016-06-01

**Authors:** Brandon J. Fast, Maria P. Galan, Ariane C. Schafer

**Affiliations:** a Dow AgroSciences LLC, Indianapolis, IN, USA; b Dow AgroSciences Sementes & Biotecnologia Brasil Ltda., Andar-Ribeirão Preto, SP, Brazil; c Dow AgroSciences Industrial Ltda., Mogi Mirim, SP, Brazil

**Keywords:** soybean (*Glycine max*), crop composition, transgenic, 2,4-D, glyphosate, glufosinate

## Abstract

Soybean event DAS-444Ø6-6 is tolerant to the herbicides 2,4-D, glyphosate, and glufosinate. An investigation of potential unintended adverse compositional changes in a genetically modified crop is required to meet government regulatory requirements in various geographies. A study to meet these requirements in Brazil was completed demonstrating compositional equivalency between DAS-444Ø6-6 and non-transgenic soybean. This study supplements the extensive literature supporting transgenesis as less disruptive of crop composition compared with traditional breeding methods.

## INTRODUCTION

The genetically modified (GM) soybean event DAS-444Ø6-6 (Enlist E3™), jointly developed by MS Technologies and Dow AgroSciences LLC, expresses the enzymes aryloxyalkanoate dioxygenase-12 (AAD-12), 5-enolpyruvylshikimate-3-phosphate synthase (2mEPSPS), and phosphinothricin acetyltransferase (PAT), which provide tolerance to the herbicides 2,4-D, glyphosate, and glufosinate, respectively. While research spanning 2 decades has indicated that the composition of GM crops is less affected compared with traditionally bred crops (Herman and Price, 2013; Schnell et al., 2014), government regulation has only become more onerous with regards to the conduct of composition studies for GM crops to meet global requirements (Paoletti and Germini, 2012). To supplement the compositional equivalency demonstrated in field trials conducted in the USA (Lepping et al., 2013), a study was also conducted in Brazil. Here we report the results of a Brazilian composition study for event DAS-444Ø6-6 that was conducted to support the regulatory approval for cultivation of this GM event in that geography.

## MATERIALS AND METHODS

Field trials were conducted at Cravinhos (SP) and Uberlândia (MG) Brazil during the 2011–2012 growing season. Test entries included soybean event DAS-444Ø6-6 (treated and not treated with 2,4-D + glyphosate) and a non-GM near-isogenic control (isoline). Three replicate blocks were included at each field site arranged in a randomized complete block design. Plots consisted of 10 rows that were 10 m long with 0.5 m row spacing. Appropriate irrigation, fertilizer, and insect, weed, and disease control were applied to the entire trial area to produce a commercially acceptable crop. The sprayed DAS-444Ø6-6 entry received 4 sequential 2,4-D + glyphosate applications (15 d before planting, at planting, V3–V4, and R1–R2) at a rate of 1170 and 1230 g ae/ha, respectively, in a spray volume of 100 L/ha. Forage samples (approximately 300 g/plot) were collected at the R3 growth stage and seed samples (approximately 500 g/plot) were collected at the R8 (maturity) growth stage. Forage samples were analyzed for proximates and minerals, and seed samples were analyzed for proximates, fiber, amino acids, fatty acids, minerals, vitamins, antinutrients, and bioactives as previously described (Fast et al., 2015; Herman et al., 2011; Lepping et al., 2013). With the exception of moisture, all values were expressed on a dry-weight basis.

Data were subjected to analysis of variance using a mixed model with entry designated as a fixed effect and location, replicate within location, and entry by location designated as random effects (SAS Institute, 2002). *T* tests were used to perform paired contrasts between DAS-444Ø6-6 and the isoline, and P-values were adjusted for multiplicity using a false discovery rate correction (Benjamini and Hochberg, 1995); results with P-values less than 0.05 were considered statistically significant. The mean composition levels of the isoline were regressed against those of the non-sprayed DAS-444Ø6-6 soybean entry for various analyte groupings (proximates and fiber, minerals, amino acids, fatty acids, vitamins, and antinutrients and bioactives) to further investigate the compositional profiles of the seed and forage. Analytes were excluded from the analysis of variance and regression plots if more than 50% of the results were less than the limit of quantitation (< LOQ).

## RESULTS

**FIGURE 1.**
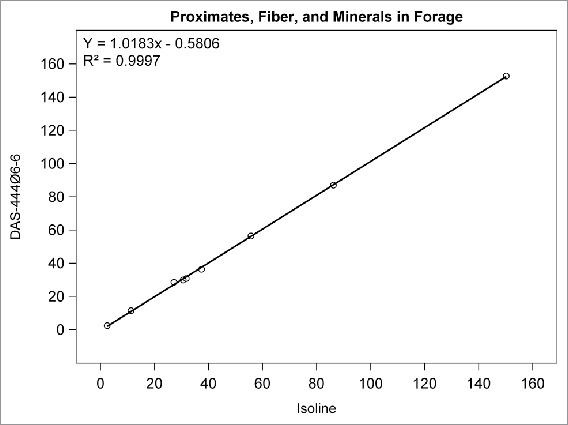
Proximates, fiber, and minerals in forage. Points from left to right: crude fat (%DW), ash (%DW), ADF (%DW), protein (%DW), NDF (%DW), phosphorus (mg/kg), carbohydrates (%DW), moisture (%FW), calcium (mg/kg).

**FIGURE 2.**
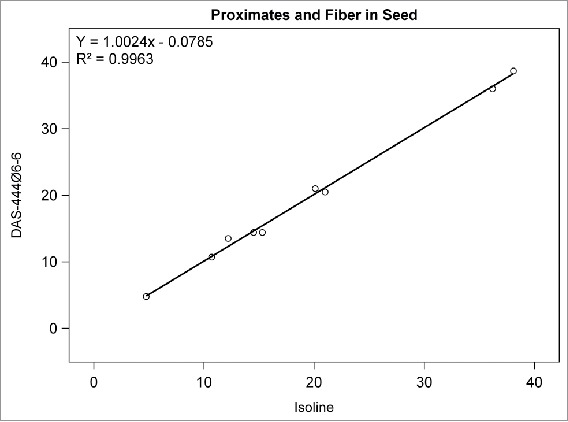
Proximates and fiber in seed. Points from left to right: ash, crude fiber, ADF, NDF, moisture, total dietary fiber, crude fat, carbohydrates, and protein (moisture = %FW, all others = %DW).

**FIGURE 3.**
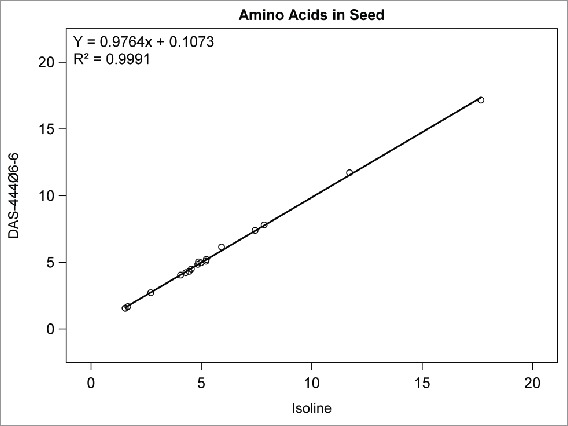
Amino acids in seed. Points from left to right: methionine, tryptophan, cystine, histidine, tyrosine, threonine, glycine, alanine, isoleucine, proline, valine, serine, phenylalanine, lysine, arginine, leucine, arpartic acid, glutamic acid (% of total amino acids).

**FIGURE 4.**
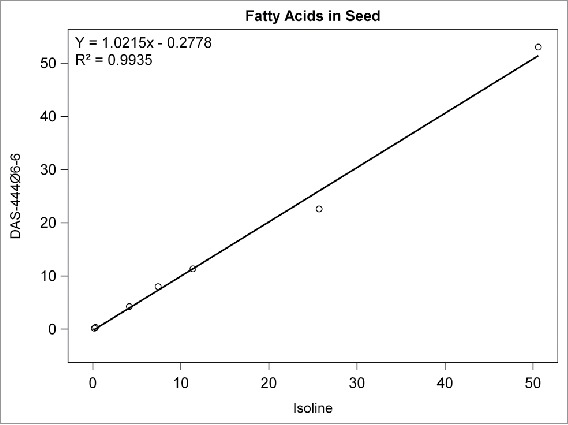
Fatty acids in seed. Points from left to right: eicosenoic, behenic, arachidic, stearic, linolenic, palmitic, oleic, and linoleic acid (% of total fatty acids).

**FIGURE 5.**
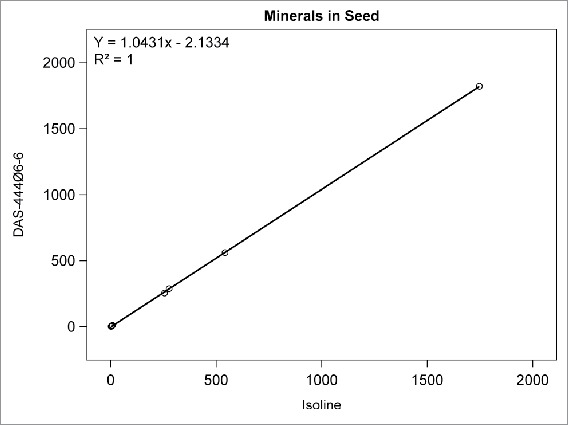
Minerals in seed. Points from left to right: copper, manganese, zinc, iron, magnesium, calcium, phosphorus, potassium (mg/100 g).

**FIGURE 6.**
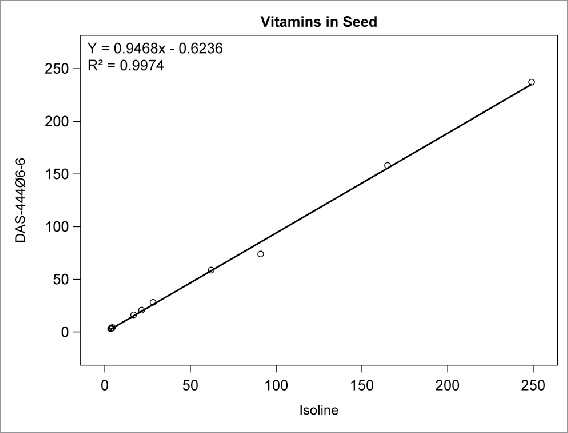
Vitamins in seed (mg/kg): Points from left to right: vitamins B_2_, B_1_, B_9_, B_6_, B_5_, alpha tocopherol, vitamin B_3_, delta tocopherol, vitamin C, gamma tocopherol, total tocopherol.

**FIGURE 7.**
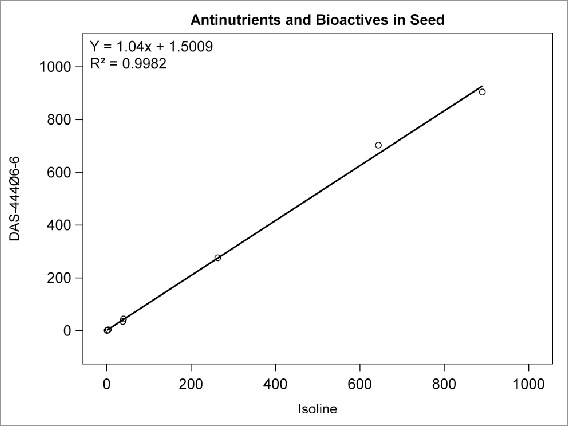
Antinutrients and bioactives in seed. Points from left to right: raffinose (%DW), phytic acid (%DW), stachyose (%DW), lectin (HU/mg), trypsin inhibitor (TIU/mg), glycitein (µg/g), daidzein (µg/g), genistein (µg/g).

Among the compositional analytes measured in DAS-444Ø6-6 and isoline soybean seed, the following analytes were found to be <LOQ: selenium, sodium; caprylic, capric, lauric, myristic, myristoleic, pentadecanoic, pentadecenoic, palmitoleic, heptadecanoic, heptadecenoic, γ-linolenic, eicosadienoic, eicosatrienoic, and arachidonic acids; and beta-tocopherol. There was no statistically significant difference between sprayed or non-sprayed DAS-444Ø6-6 soybean and the non-GM isoline for any of the remaining 71 compositional components measured in forage and seed (data not shown). The composition levels in the isoline were regressed against those of the non-sprayed DAS-444Ø6-6 soybean entry for various analyte groupings to further investigate the compositional profiles of the seed and forage (Figs. 1-7). In all cases, the coefficient of determination for these relationships was greater than 0.99. Additionally, the slopes of the regression lines were very close to one (0.9468−1.0431), and all data points clustered close to the regression lines. This analysis was also done for logarithm base-10 transformed data to help mitigate weighting that might occur due to the distribution of certain points in the natural scale (data not shown). In this latter analysis the coefficient of determination was also greater than 0.99 in all cases, and the slopes ranged from 0.997 to 1.011. These diagnostics reinforce the equivalency between the composition profiles of DAS-444Ø6-6 and the isoline.

## DISCUSSION

The use of regression plots to present mean results allows the results for numerous analytes to be visually represented within a small number of plots compared with the lengthy results tables that are required to present results from t tests comparing DAS-444Ø6-6 with the isoline; in this research, results from 71 analytes are presented in 7 plots. The results of this research demonstrate the compositional equivalency of event DAS-444Ø6-6 and isoline soybean seed, and the regression plots used to present the results help reinforce the equivalency through visual representation of the data. Furthermore, these results add to the empirical evidence developed over the last 20 y that supports the underlying mechanistic understanding of the genetic mechanisms that cause unintended compositional changes and their prediction that trait introgression via transgenesis is less disruptive of crop composition compared with traditional breeding techniques (Herman and Price, 2013; Ricroch et al., 2011; Schnell et al., 2014).

## DISCLOSURE OF POTENTIAL CONFLICTS OF INTEREST

The authors declare the following competing financial interest(s): BF and MG are employed by Dow AgroSciences, which develops and markets transgenic seed. AS was formerly employed by Dow AgroSciences and is currently employed by Covance Laboratories, which was contracted by Dow AgroSciences to conduct the composition analytical portion of this study.

## References

[cit0008] Benjamini Y, Hochberg Y Controlling the false discovery rate - a practical and powerful approach to multiple testing. Journal of the Royal Statistical Society Series B-Methodological 1995; 57:289-300.

[cit0005] Fast BJ, Schafer AC, Johnson TY, Potts BL, Herman RA. Insect-protected event DAS-81419-2 soybean (Glycine max L.) grown in the United States and Brazil is compositionally equivalent to nontransgenic soybean. J Agric Food Chem 2015; 63:2063-73; PMID:25641393; http://dx.doi.org/10.1021/jf505015y 25641393 PMC4342727

[cit0006] Herman RA, Phillips AM, Lepping MD, Sabbatini J Compositional safety of DAS-68416-4 (AAD-12) herbicide-tolerant soybean. J Nutr Food Sci 2011; 1:2; http://dx.doi.org/10.4172/2155-9600.1000103

[cit0002] Herman RA, Price WD. Unintended compositional changes in Genetically Modified (GM) crops: 20 years of research. J Agric Food Chem 2013; 61:11695-701; PMID:23414177; http://dx.doi.org/10.1021/jf400135r 23414177

[cit0004] Lepping MD, Herman RA, Potts BL. Compositional equivalence of DAS-444Ø6-6 (AAD-12 + 2mEPSPS + PAT) herbicide-tolerant soybean and nontransgenic soybean. J Agric Food Chem 2013; 61:11180-90; PMID:24191699; http://dx.doi.org/10.1021/jf403775d 24191699

[cit0003] Paoletti C, Germini A Compositional analysis: field trial design and statistical analysis. EFSA technical meeting with applicants on GMOs. Parma 2012.

[cit0009] Ricroch AE, Berge JB, Kuntz M. Evaluation of genetically engineered crops using transcriptomic, proteomic and metabolomic profiling techniques. Plant Physiol 2011; 155:1752-61; PMID:21350035; http://dx.doi.org/10.1104/pp.111.173609 21350035 PMC3091128

[cit0007] SAS Institute Inc SAS/STAT® 9.3 user's guide Cary, NC: SAS Institute, 2011.

[cit0001] Schnell J, Steele M, Bean J, Neuspiel M, Girard C, Dormann N, Pearson C, Savoie A, Bourbonnière L, Macdonald P. A comparative analysis of insertional effects in genetically engineered plants: considerations for pre-market assessments. Transgenic Res 2014; 24:1-17; PMID:25344849 25344849 10.1007/s11248-014-9843-7 PMC4274372

